# Randomized phase II study of anastrozole plus tegafur-uracil as neoadjuvant therapy for ER-positive breast cancer in postmenopausal Japanese women (Neo-ACET BC)

**DOI:** 10.1007/s00280-018-3544-5

**Published:** 2018-02-21

**Authors:** Takahiro Nakayama, Yasuaki Sagara, Tsutomu Takashima, Nobuki Matsunami, Norikazu Masuda, Yasuo Miyoshi, Tetsuya Taguchi, Toyokazu Aono, Toshikazu Ito, Tatsuo Kagimura, Shinzaburo Noguchi

**Affiliations:** 1 Department of Breast and Endocrine Surgery, Osaka International Cancer Institute, 3-1-69, Otemae, Chuou, Osaka, 541-8567 Japan; 2 Department of Breast Surgery, Sagara Hospital, Kagoshima, Japan; 3 Department of Surgical Oncology, Osaka City University Hospital, Osaka, Japan; 4 Department of Surgery, Osaka Rosai Hospital, Sakai, Japan; 5 Department of Surgery, Breast Oncology, National Hospital Organization Osaka National Hospital, Osaka, Japan; 6 Department of Surgery, Division of Breast and Endocrine Surgery, Hyogo College of Medicine, Nishinomiya, Japan; 7 Department of Endocrine and Breast Surgery, Kyoto Prefectural University of Medicine, Kyoto, Japan; 8 Department of Breast Surgery, Osaka Breast Clinic, Osaka, Japan; 9 Department of Surgery, Rinku General Medical Center, Izumisano, Japan; 10 Translational Research Informatics Center, Foundation for Biomedical Research and Innovation, Kobe, Japan; 11 Department of Breast and Endocrine Surgery, Graduate School of Medicine Osaka University, Suita, Japan

**Keywords:** Breast cancer, Anastrozole, UFT, Clinical study, Neoadjuvant therapy

## Abstract

**Purpose:**

This phase II study evaluated the efficacy and safety of anastrozole concurrent with tegafur/uracil (UFT) as neoadjuvant therapy for ER-positive postmenopausal breast cancer.

**Methods:**

Postmenopausal Japanese women with ER-positive, HER2-negative, T2,N0-1,M0 breast cancer seen at tertiary hospitals were eligible for this open-label, randomized, multicenter study. Patients were randomized 1:1 by minimization to orally receive either anastrozole (1 mg once daily) plus UFT (tegafur/uracil combination in 1:4 molar ratio; 270 mg/m^2^/day in two divided doses) or anastrozole (as above) alone for 24 weeks. Tumor response was assessed by investigator and central review as per RECIST v1.1. The primary endpoint was the proportion of patients with best overall response of CR or PR [clinical response rate (RR)] determined by central radiologic review.

**Results:**

The study was prematurely terminated due to Grade ≥ 3 liver dysfunction reported in 3 patients receiving anastrozole/UFT. Of 57 patients randomized before termination (29 anastrozole/UFT, 28 anastrozole), all were analyzed for safety and 56 (28 each group) for tumor response. Compared with anastrozole alone, anastrozole/UFT did not achieve significantly higher RR [39.3% (90% CI 23.8–56.5%) vs 14.3% (90% CI 5.0–29.8%); *p* = 0.0683, Fisher’s exact test], but produced significantly greater tumor shrinkage (mean tumor reduction rate 31.0 vs. 14.2%; *p* = 0.0181, unpaired *t*-test). Grade ≥ 3 adverse events were more common with anastrozole/UFT than with anastrozole (17.2 vs. 0%).

**Conclusion:**

Although the study was terminated owing to the altered liver function, it showed that there was a trend to greater shrinkage of tumor in the combination group for ER-positive, HER2-negative postmenopausal breast cancer.

## Introduction

Breast cancer, even at its early stage, often presents with disseminated micrometastases, which cannot be adequately controlled by locoregional treatments based on surgery and radiotherapy. Neoadjuvant and adjuvant systemic chemotherapy and endocrine therapy have been proposed to eradicate these micrometastases [1–3]. Neoadjuvant chemotherapy, which has been the mainstay of neoadjuvant medical breast cancer treatment, has proven benefits, such as increasing the likelihood of breast conservation and improving the long-term prognosis in patients achieving pathological complete response (pCR). Of note, some investigators have reported similar long-term prognosis of breast cancer patients after neoadjuvant chemotherapy to that after adjuvant (postoperative) chemotherapy [4]. Factors known to affect the breast cancer response to neoadjuvant chemotherapy include hormone-receptor (HR) status and human epidermal growth factor receptor 2 (HER2) status of the tumor. In patients with estrogen receptor (ER)-positive, HER2-negative tumors, neoadjuvant chemotherapy has limited activity, rarely causing pCR, and has not been shown to improve the long-term prognosis [5, 6]. Neoadjuvant endocrine therapy with an aromatase inhibitor (AI) has emerged as a newer strategy for breast cancer of this phenotype [7]. A randomized controlled study has shown comparable efficacy of neoadjuvant AI versus chemotherapy for ER-positive, HER2-negative, postmenopausal breast cancer with regard to pCR and breast conservation rates, but significantly less adverse events with AI than with chemotherapy [8]. However, breast cancers with aggressive clinical features such as Luminal B subtype are less responsive to endocrine treatments, showing poor response even to neoadjuvant AI. Regarding cytotoxic agents, metronomic dosing has been devised to avoid their dose-dependent toxicities and to sustain their effects, so as to produce antiangiogenesis that can indirectly lead to an inhibition of tumor cell proliferation. Metronomic therapy with cyclophosphamide and UFT (tegafur/uracil combination in a fixed molar ratio of 1:4) has gained wide acceptance [9, 10]. Anastrozole administered in combination with UFT exhibited significantly higher antitumor activity as compared with either agent alone against a human breast cancer cell line grown in mice [11], suggesting promising activity of endocrine therapy concurrent with metronomic chemotherapy for breast cancer in humans.

With these backgrounds, we conducted this randomized phase II study to evaluate anastrozole plus UFT as neoadjuvant therapy for HR-positive breast cancer in postmenopausal Japanese women.

## Materials and methods

### Study design

This was an open-label, randomized, multicenter, phase II study designed to evaluate the efficacy and safety of anastrozole plus UFT versus anastrozole alone as neoadjuvant therapy for ER-positive, HER2-negative breast cancer in postmenopausal Japanese women. The study was approved by the institutional review board of each study site and was conducted in accordance with the Declaration of Helsinki and ethical principles of clinical investigations. Prior to enrollment, all patients provided informed consent to the study. The study has been registered with UMIN-CTR (UMIN000006434).

### Patients and treatment

Eligible patients were postmenopausal women with ER-positive (≥ 10%), HER2-negative (immunohistochemistry staining score 0 or 1+, or fluorescence in situ hybridization negative), T2,N0-1,M0 invasive ductal or lobular carcinoma of breast who had an Eastern Cooperative Oncology Group (ECOG) performance status (PS) score of 0 or 1. Patients were centrally enrolled and randomized 1:1 to receive anastrozole plus UFT or anastrozole alone. Randomization was done by the minimization method following stratification with respect to extent of lymph node involvement (N0 or N1) and progesterone receptor (PgR) status of the tumor (positive, negative or unknown).

Patients allocated to the anastrozole alone group were given oral anastrozole at 1 mg once daily for 24 weeks, whereas those allocated to the anastrozole plus UFT group were given oral anastrozole at the same dosage in addition to oral UFT at 270 mg/m^2^/day (recommended daily dose per body surface area at enrollment) in two divided doses for 24 weeks. Throughout the treatment period, patients in both groups were prohibited to receive any non-study treatment for breast cancer (e.g., hormones other than anastrozole, cytotoxic agents, radiotherapy, and biological response modifiers). Patients in the anastrozole/UFT group were also prohibited to receive any antineoplastic agent other than UFT. All patients were to undergo surgery at 1–4 weeks after the end of the protocol neoadjuvant treatment.

### Endpoints and statistical analysis

The primary endpoint was the proportion of patients with best overall response of CR or PR (clinical response rate [RR]) determined by central radiologic review as per RECIST v1.1. A tumor lesion with larger diameter ≥ 10 mm as measured by MRI (preferred) or CT and ≥ 10 mm as measured using a caliper was defined as measurable. Tumor size was measured by MRI or CT at the start and the end of the protocol treatment and using a caliper and ultrasonography every 4 weeks during the treatment. Overall response was determined every 4 weeks based on combined assessment of the response of target lesion(s) and appearance of any new lesion, and the best of the overall responses seen during the treatment period was defined as best overall response. Secondary endpoints included percent changes in tumor diameter measured by MRI or CT from baseline to the end of treatment for individual patients (tumor reduction rate), histological tumor response, and proportion of patients undergoing breast conserving surgery (breast conservation rate) as well as severity and frequency of adverse events (AEs). Histological tumor response was assessed according to criteria adapted from the General Rules for Clinical and Pathological Recording of Breast Cancer ver16 (2008), and the proportions of patients showing Grade ≥ 1b response was calculated. AEs were coded and graded using the CTCAE v4.0.

By reference to RR values previously obtained with neoadjuvant anastrozole using caliper-based tumor size measurements [12, 13], the threshold RR was assumed to be 45%. The least number of subjects needed to show an expected RR of 65% for anastrozole plus UFT with a one-sided alpha error rate of 5% and a statistical power of 90% was calculated as 53. Hence, a sample size of 120 (60 per group) was planned by taking into account the potential of excluding ineligible subjects.

The efficacy analysis set was composed of all randomized patients but those who proved to be ineligible after randomization. The safety analysis set was composed of all randomized patients but those who received no doses of the protocol treatment assigned.

For RR (primary endpoint), the 90% confidence interval (CI) was calculated to test whether the lower bound of the 90% CI for the RR obtained with anastrozole plus UFT was at or above the threshold RR (45%). In an exploratory manner, RR data were compared between the two groups using Fisher’s exact test. Among secondary endpoints, tumor reduction rates were compared between the two groups using the unpaired *t*-test. All tests were two-sided, and a *p* value less than 0.05 was considered statistically significant.

## Results

Accrual was suspended on March 27, 2012, when 57 patients had been enrolled because of serious liver dysfunction [increased alanine aminotransferase (ALT) and increased aspartate aminotransferase (AST)] reported in 3 of 29 patients assigned to the anastrozole plus UFT group by this date. Subsequently, the data center performed an interim analysis of data from the 57 patients. The independent data monitoring board concluded that the results of the analysis did not warrant continuation of the study and recommended the protocol committee to terminate the study. Following the recommendation, the committee decided to terminate the study on February 19, 2014.

### Patient characteristics

**Fig. 1:**
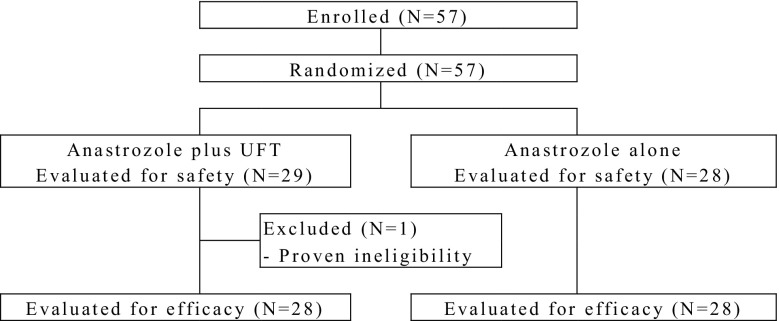
Subject disposition

All 57 patients enrolled between December 1, 2010 and March 27, 2012 were randomized to receive either anastrozole plus UFT (*n* = 29) or anastrozole alone (*n* = 28). All of them were evaluated for safety, while 28 in each group were evaluated for tumor response (Fig. 1). The patient randomized to the anastrozole plus UFT group and excluded from the efficacy analysis set proved to be ineligible after randomization. The two treatment groups were well-matched with respect to baseline characteristics (Table 1).

**Table 1:** Patient characteristics

*N* (%)	Anastrozole plus UFT (*N* = 29)	Anastrozole alone (*N* = 28)	*P* value*
Age [years], median (range)	70 (51–87)	65.5 (50–81)	0.0901
BSA [m^2^], median (range)	1.5 (1.3–1.9)	1.5 (1.3–1.9)	0.0935
*N* status			1.0000
N0	21 (72.4)	20 (71.4)	
N1	8 (27.6)	8 (28.6)	
PgR‒IHC			1.0000
Negative	8 (27.6)	8 (28.6)	
Positive	21 (72.4)	20 (71.4)	
Histological subtypes			1.0000
Invasive carcinoma of no special type	27 (93.1)	26 (92.9)	
Special subtypes (invasive lobular carcinoma)	2 (6.9)	2 (7.1)	
Form of surgery offered at presentation			0.2829
Breast conserving surgery	15 (51.7)	19 (67.9)	
Mastectomy	14 (48.3)	9 (32.1)	
PS (ECOG)			1.0000
0	27 (93.1)	26 (92.9)	
1	2 (6.9)	2 (7.1)	

*BSA* body surface area, *ECOG* Eastern Cooperative oncology group, *IHC* immunohistochemistry, *PgR* progesterone receptor, *PS* performance status

*Wilcoxon rank sum test or Fisher’s exact test

### Efficacy

As shown in Table 2, the anastrozole plus UFT group had RR (90% CI) of 39.3% (23.8–56.5%) with two CRs (7.1%) and 9 PRs (32.1%); the lower bound of the 90% CI was less than the threshold RR (45%). Thirteen patients (46.4%) had best overall response of SD. The RR in the experimental group was numerically but not significantly higher than that in the control group [14.3% (90% CI 5.0–29.8%); *p* = 0.0683, Fisher’s exact test]. In the experimental and control groups, two versus zero patients achieved CR, while no versus 3 patients experienced PD. Of 25 and 27 patients evaluable for histological tumor response in the respective groups, 8 (32.0%) and 12 (44.4%) patients showed Grade ≥ 1b histological response as per the criteria adapted from the General Rules for Clinical and Pathological Recording of Breast Cancer ver16.

**Table 2:**
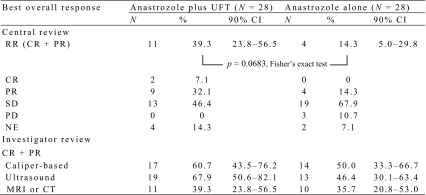
Clinical response rate (RR)

**Fig. 2:**
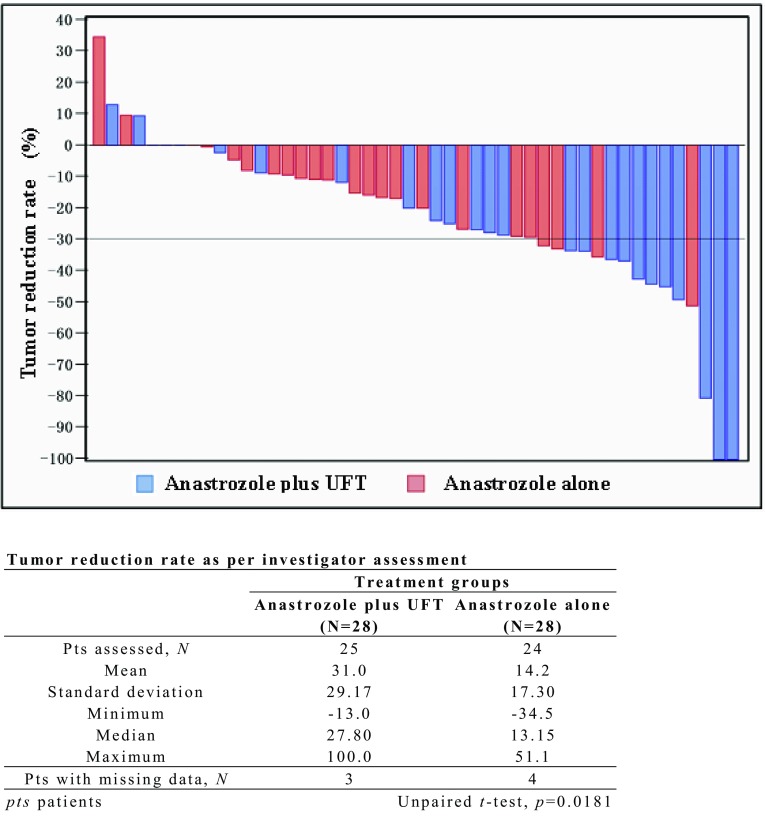
Waterfall plot of tumor reduction rate (percent reduction in unidimensional radiologic measurements)

Figure 2 shows a waterfall plot of tumor reduction rate. As compared with anastrozole alone, anastrozole plus UFT produced significantly greater tumor shrinkage with the mean (SD) tumor reduction rate being 31.0 (29.17) % versus 14.2 (17.30) % (*p* = 0.0181, unpaired *t*-test).

After receiving the protocol treatment, 25 patients in the anastrozole plus UFT group and 28 in the anastrozole alone group were offered surgery. Of these, 19 [76.0% (95% CI 54.9–90.6%)] and 16 [57.1% (95% CI 37.2–75.5%)] patients underwent breast conserving surgery.

### Safety

AEs occurred in 22/29 patients (75.9%) in the anastrozole plus UFT group versus 17/28 patients (60.7%) in the anastrozole alone group (*p* = 0.2630, Fisher’s exact test). Grade ≥ 3 AEs were more frequent in the anastrozole plus UFT group (*n* = 5, 17.2%) than in the anastrozole alone group (*n* = 0, 0%) (*p* = 0.0518, Fisher’s exact test). The most common AEs in the anastrozole plus UFT group included increased ALT (55.2%), increased AST (48.3%), increased blood bilirubin (41.4%), thrombocytopenia (31.0%), and fatigue (31.0%) (Table 3). Grade 3 AEs were increased AST (10.3%), increased ALT (10.3%), back pain (3.4%), and hypertriglyceridemia (3.4%). No Grade ≥ 3 AEs were reported in the anastrozole alone group. No deaths occurred in either group.

**Table 3:** Adverse events collected in a solicited manner

Events, *N* (%)	Anastrozole plus UFT (*N* = 29)		Anastrozole alone (*N* = 28)	
	Any grade	Grade ≥ 3	Any grade	Grade ≥ 3
Alanine aminotransferase increased	16 (55.2)	3 (10.3)	6 (21.4)	0
Aspartate aminotransferase increased	14 (48.3)	3 (10.3)	4 (14.3)	0
Blood bilirubin increased	12 (41.4)	0	0	0
Thrombocytopenia	9 (31.0)	0	1 (3.6)	0
Fatigue	9 (31.0)	0	2 (7.2)	0
Anorexia	7 (24.1)	0	0	0
Nausea	7 (24.1)	0	0	0
Leukopenia	5 (17.2)	0	4 (14.3)	0
Diarrhea	4 (13.8)	0	4 (14.3)	0
Creatinine increased	3 (10.3)	0	3 (10.7)	0
Skin hyperpigmentation	3 (10.3)	–	0	–
Hemoglobin increased	2 (6.9)	0	1 (3.6)	0
Mucositis oral	2 (6.9)	0	2 (7.2)	0
Vomiting	1 (3.4)	0	0	0
Rash maculopapular	1 (3.4)	0	1 (3.6)	0

## Discussion

In this randomized phase II study, we evaluated the efficacy and safety of anastrozole plus UFT versus anastrozole alone as neoadjuvant therapy for ER-positive, HER2-negative postmenopausal breast cancer.

Although the study was terminated owing to the altered liver function, it showed there was a trend to greater shrinkage of tumor in the combination group.

In general, tumor cells with higher proliferative activity are more sensitive to chemotherapy. Endocrine therapy inhibits the proliferation of tumor cells and therefore counteracts the cytotoxic activity of chemotherapy used concomitantly [14]. In contrast, metronomic chemotherapy has been suggested to enhance the antitumor activity of endocrine therapy, as it inhibits tumor growth through antiangiogenesis. Previous studies have demonstrated a benefit of 2-year postoperative adjuvant therapy with UFT concurrent with tamoxifen for ER-positive breast cancer [15–18]. In the present study, anastrozole plus UFT produced a higher tumor reduction rate as compared with anastrozole alone.

During this combination therapy, 2 patients achieved CR and no patients had best overall response of PD. The former finding suggests that the addition of UFT may enhance the activity of endocrine therapy, while the latter finding reflects the activity of UFT against hormone-resistant tumor cells potentially existing in an HR-positive breast tumor. Thus, the combination of UFT with anastrozole may have conferred broad-spectrum antitumor activity that could cover hormone-sensitive as well as hormone-resistant tumors.

This study failed to show that the lower bound of the 90% CI for the RR in the anastrozole plus UFT group was at or above the threshold RR (45%). We used radiological (MRI or CT) tumor measurements to determine the RR (primary endpoint) despite the use of previous response data obtained by caliper-based measurements to set the threshold RR. Radiological response assessments are generally worse than caliper-based assessments, as was observed in the present study [RR 37.5% (21/56) vs 55.4% (31/56) as per investigator assessment]. This might partially account for the failure of the study to meet its primary endpoint. The RR determined by caliper-based measurements in patients treated with anastrozole plus UFT was 60.7% (17/28), which is comparable to the expected value (65%) and deserves evaluation.

While anastrozole plus UFT tended to produce greater clinical tumor response as compared with anastrozole alone, histological tumor response showed an opposite tendency. This might be partly due to a great inter-site, inter-rater variability in ratings of histological tumor response to neoadjuvant endocrine therapy, which is likely to occur when many of the pathologists involved have little experience in this rating.

Grade ≥ 3 liver dysfunction occurred in 3 patients (10.3%) in the anastrozole plus UFT group. All events resolved and none of them resulted in death. Although a previous special drug-use survey reported similar frequency of liver dysfunction between patients treated with UFT plus anastrozole and those treated with UFT alone [19], the occurrence of severe liver dysfunction after concurrent treatment with anastrozole and UFT in the present study indicates the need for careful monitoring of liver function during this combination therapy in practice.

This study had several limitations. First, the study was probably underpowered, because it was terminated before achieving the planned sample size (*n* = 120). Second, no patients enrolled in this study were followed up for survival after years, because the association of achievement of pCR after neoadjuvant treatment with a better long-term outcome remained controversial. Third, this study was conducted with no regard to classification by risk factors; luminal breast cancer with high-risk features (e.g., with a high Ki67 index) may be sensitive to chemotherapy.

Furthermore, the optimal duration of neoadjuvant therapy for breast cancer has not been established. In the present study, patients were to receive neoadjuvant endocrine or chemoendocrine therapy for 24 weeks (6 months). Previous studies have reported a higher breast conservation rate after 6-month versus 4-month neoadjuvant therapy [20], achievement of maximal tumor response after 6- to 12-month neoadjuvant therapy [21], and a significantly higher tumor response rate after 12 months than a shorter period of neoadjuvant therapy [22]. In the N-SAS BC06 study, the majority of postmenopausal Japanese women with breast cancer scheduled to undergo mastectomy could undergo breast conserving surgery after receiving neoadjuvant therapy for 6 months [23]. To increase the likelihood of breast conservation, neoadjuvant therapy should be continued for at least 3 to 4 months, preferably for up to 6 months, as long as the patient shows no disease progression during the therapy.

Recently, a cyclin-dependent kinase (CKD) 4/6 inhibitor has emerged as a promising new drug for HR-positive breast cancer. The CKD 4/6 inhibitor in combination with an endocrine agent has been shown to significantly prolong progression-free survival as compared with the endocrine agent alone in advanced breast cancer [24–26], and this effect was reported to be independent of the degree of endocrine resistance, hormone-receptor expression level, and PIK3CA mutational status [26].

In conclusion, although the study was terminated owing to the altered liver function, it showed there was a trend to greater shrinkage of tumor in the combination group. The combination of an AI with an oral fluoropyrimidine may become an attractive option as neoadjuvant therapy for ER-positive, HER2-negative breast cancer. A study is currently underway to evaluate the benefit of endocrine therapy combined with S-1 as adjuvant (postoperative) therapy for ER-positive, HER2-negative breast cancer (POTENT study). The results of this ongoing study will further clarify the clinical role of adding metronomic chemotherapy to endocrine therapy for this phenotype of breast cancer.
